# Risk Factors Associated with Acute Pancreatitis after Percutaneous Biliary Intervention: We Do Not Know Nearly Enough

**DOI:** 10.1155/2023/9563074

**Published:** 2023-01-06

**Authors:** Jing Song, Jun Deng, Feng Wen

**Affiliations:** Department of Radiology, Shengjing Hospital of China Medical University, Shenyang 110004, China

## Abstract

Percutaneous transhepatic cholangiodrainage (PTCD) and percutaneous transhepatic biliary stenting (PTBS) may be used as a palliative treatment for inoperable patients with malignant biliary obstruction (MBO) to improve the prognosis and their quality of life. However, acute pancreatitis is a common and severe complication that cannot be ignored after PTCD and PTBS in patients with MBO. A few cases may develop severe pancreatitis with a higher mortality rate. In this study, we summarize the known risk factors for acute pancreatitis after percutaneous biliary interventional procedures and investigate possible risk factors to reduce its occurrence by early identifying high-risk patients and taking appropriate measures.

## 1. Introduction

Malignant biliary obstruction (MBO) is usually due to endogenous obstruction or exogenous compression of the bile duct. Obstruction may arise from pancreatic cancer, bile duct cancer, cancer of Vater's ampulla [1–3], other metastatic tumors, or malignant lymphadenopathy [4, 5]. The clinical manifestations are mainly cholestatic jaundice, cutaneous pruritus, and abnormal hepatic function [6]. However, most patients are in an advanced stage of the tumor (peripheral vessel involvement and poor general condition) at the time of diagnosis and are unsuitable for curative resection [7]. With the development and maturity of interventional technology, percutaneous transhepatic cholangiodrainage (PTCD) and percutaneous transhepatic biliary stenting (PTBS) have become the primary palliative therapy for some patients with MBO. This treatment may prolong survival and alleviate liver failure caused by obstructive jaundice. Various complications may develop after PTCD and PTBS, including acute biliary infections, acute pancreatitis, hemobilia, and in-stent restenosis [8]. Among these, acute pancreatitis is a relatively common complication with an incidence of approximately 0.9–24.2% [9–13] and seriously affects the prognosis of patients with MBO. Previous studies have investigated the risk factors for acute pancreatitis after endoscopic retrograde cholangiopancreatography (ERCP) [14–17]. However, the research on the clinical risk factors of acute pancreatitis after percutaneous biliary intervention is limited, and the pathogenesis remains unclear.

In this study, we attempted to summarize and analyze the risk factors of acute pancreatitis after percutaneous interventional therapy in previous studies and discuss possible associated factors. Furthermore, we compared risk factors for different causes of pancreatitis, such as common acute pancreatitis, post- ERCP pancreatitis (PEP), and acute pancreatitis after percutaneous interventional therapy. We would like some suggestions for preventing and treating acute pancreatitis after percutaneous biliary interventional procedure.

## 2. Known Risk Factors

### 2.1. Patient-Related Factors

Regarding inherent patient factors, the previous studies mainly focused on the condition of the pancreas itself. The exocrine glands of the pancreas are composed of acinar and glandular duct cells. The acinar cells synthesize and secrete digestive enzymes and finally drain into the duodenum through the pancreatic duct [18, 19]. Some research showed that acute pancreatitis after the percutaneous biliary intervention was closely related to the status of the exocrine glands of the pancreas, including the pancreatic parenchyma and the main pancreatic duct.

#### 2.1.1. Abundant Pancreatic Parenchyma

Previous studies defined pancreatic atrophy when the parenchyma thickness was <10 mm [20]. However, existing clinical studies suggested that pancreatic parenchymal atrophy was a protective factor for acute pancreatitis after PTCD and PTBS. A retrospective study by Sugawara et al. found that the incidence of grade 3 acute pancreatitis and the increasing rate of serum amylase after placement of biliary stents in patients with atrophic pancreas were significantly lower than those in patients with the non-atrophic pancreas (7.5% vs. 36.4%, *p* = 0.004; 2.2 times vs. 11.0 times, *p* = 0.001) [9]. Li et al. found that pancreatic atrophy (OR = 0.12) was a protective factor for acute pancreatitis in pancreatic cancer patients with obstructive jaundice after PTCD [21].

The findings of the above studies were consistent with the PEP-related clinical studies. Umemura et al. investigated the influencing factors of PEP after self-expandable metallic stent (SEMS) insertion across the papilla of Vater. The results demonstrated that the incidence of PEP among patients whose thickness of the pancreatic parenchyma at the left side of the corpus vertebrae was less than 9.5 mm (0%) on computed tomography (CT) was lower than that in patients whose thickness was 9.5 mm or greater (34.4%) [22]. Takeda et al. found that a high pancreatic volume index (pancreatic volume index ≥24 mm [OR = 5.12; *p* = 0.02]) was an independent risk factor for pancreatitis after endoscopic SEMS implantation [23].

The status of the pancreatic parenchyma may affect the incidence rates of acute pancreatitis after percutaneous biliary intervention or endoscopic procedures. This is due to differences in the number of pancreatic exocrine (acini) cells [24]. At the same time, the mechanism of acute pancreatitis also involves the activation of trypsin in pancreatic acinar cells, which may be due to the stimulation of pancreatic duct epithelial and pancreatic acinar cells induced by endoscopic retrograde manipulation. The obstruction of the pancreatic duct after the biliary procedure will limit the excretion of pancreatic juice into the duodenum for some patients with abundant pancreatic parenchyma (acinar cells). If the pancreatin in pancreatic juice is activated in the pancreas, it will cause auto-digestion, edema, hemorrhage, and even necrosis of the pancreatic tissue, resulting in acute pancreatitis. In contrast, because of the exocrine insufficiency in pancreatic atrophy, the small amount of pancreatic juice secreted by the atrophic pancreatic parenchyma reduces the risk and severity of acute pancreatitis, despite the obstruction of the pancreatic duct following the procedure [14]. In addition, a study by Nakamura et al. showed a significant association (*p* < 0.001) between pancreatic exocrine function and CT-measured pancreatic parenchyma thickness [25]. Therefore, the thickness of the pancreatic parenchyma measured by a CT scan indirectly evaluates the state of the pancreatic parenchyma and then predicts the risk of acute postoperative pancreatitis.

#### 2.1.2. Main Pancreatic Duct Patency

In patients with main pancreatic duct patency, PTCD/PTBS may affect the excretion of pancreatic juice and further lead to acute pancreatitis. In a retrospective study on PTBS across Vater's papilla, Sugawara et al. found that grade 3 acute pancreatitis and serum amylase elevation in patients with main pancreatic duct obstruction were significantly lower than those with main pancreatic duct patency (12.5% vs. 36.2%, *p* = 0.026; 2.2 times vs. 12.2 times, *p* < 0.001) [9].

Additionally, the main pancreatic duct is closely involved in tumor genesis and progression for most pancreatic cancers, resulting in various extents of obstruction of the main pancreatic duct and atrophy of the distal pancreas [14, 26]. In a recent study by Kim et al., the incidence of acute pancreatitis after PTBS in patients with unresectable pancreatic cancer was only 6.4% (9/140 cases), and no statistically significant risk factors were found [10]. Previous studies on PEP showed that the type of primary tumor except pancreatic cancer was a risk factor for pancreatitis. Kawakubo et al. found that non-pancreatic cancer (mainly cholangiocarcinoma and lymph node metastasis) was a significant risk factor for acute pancreatitis after endoscopic SEMS placement (OR = 5.52; 95% CI, 2.24–14.1; *p* < 0.001) [14]. Shimizu et al. indicated that non-pancreatic cancer (OR = 3.43; 95% CI, 1.44–10.05; *p* = 0.010) was an independent risk factor of PEP [26]. Therefore, many researchers believed that percutaneous biliary intervention or endoscopic procedures were relatively safe in patients with pancreatic cancer. However, further studies would be needed for patients with non-pancreatic cancer to reduce the incidence of acute pancreatitis while focusing on the risk factors.

Generally, it is considered normal that the diameter of the main pancreatic duct is less than 3 mm [19]. Main pancreatic duct dilatation is more common in patients with pancreatic cancer, ampullary cancer, or chronic pancreatitis, often manifesting as main pancreatic duct hypertension. Thus, these patients can tolerate the increased pressure in the main pancreatic duct caused by biliary-related procedures and are less prone to postoperative pancreatitis [27]. Li et al. found that patients with pancreatic duct dilatation (OR = 0.14) had a lower probability of developing pancreatitis after PTCD, which demonstrated that placement of internal and external drainage catheters had little effect on pancreatic juice discharge in these patients [21]. A similar study reported that the main pancreatic duct dilation was a negative predictor of postoperative pancreatitis and was associated with a lower risk of PEP (*p* < 0.001) [27]. The results of a retrospective study showed that the probability of acute postoperative pancreatitis decreased by 3.91 times for every 1 mm increase in the diameter of the main pancreatic duct (95% CI, 1.23–12.4; *p* = 0.02) [22].

Thus, whether the main pancreatic duct dilation is caused by pancreatic cancer or other reasons, the patency of the main pancreatic duct directly affects the occurrence and degree of pancreatitis after percutaneous biliary intervention or endoscopic operation (especially across Vater's papilla).

### 2.2. Procedure-Related Factors

#### 2.2.1. Infectious Bile or Intestinal Fluid Reflux in the Pancreatic Duct

Previous studies reported that 30–50% of patients with MBO were complicated with biliary infections before the operation [28]. Biliary obstruction leads to impairment of bile drainage, increased pressure in the bile duct, and cholestasis causes bacterial multiplication. Moreover, the incidence and severity of biliary tract infection increase with the duration of obstruction. Furthermore, biliary tract infection is a common complication after biliary interventional therapy. Rösch et al. reported that the positive rate of bacteria in the bile after PTCD increased from 60 to 100%, and 30% of the patients developed cholangitis [29]. A previous study showed that the early intestinal-biliary reflux was obstructed after placing a metal stent across the ampulla [30].

This kind of acute pancreatitis because of infection can be caused by infected bile or reflux of intestinal fluid. Previous studies indicated that injection of sterile bile into the pancreatic duct did not damage pancreatic tissue, but infected bile could cause acute pancreatitis [31, 32]. The reasons for this pancreatitis are, on the one side, dysfunction or non-function of the sphincter of Oddi, and on the other side, increased pressure in the biliary tract or intestine. The compressed cholangiography, inadequate drainage of cholestatic bile, or placement of internal drainage catheters and stents across the ampulla may result in the loss of Oddi's sphincter function. In this situation, the infected bile or intestinal fluid can flow back into the pancreatic duct, activate pancreatic enzymes, damage the pancreatic duct mucosa and pancreas, cause pancreatic inflammatory edema, and induce pancreatitis.

Therefore, appropriate anti-inflammatory treatment should be performed for patients with biliary tract infections before the operation. The strict aseptic operation, adequate drainage of the bile, and avoidance of pressurized cholangiography should be performed during the interventional procedure. The placement of biliary stents across the sphincter of Oddi should be avoided to prevent the infection of intestinal fluid reflux. For patients with biliary tract infections who plan to implant stent across the ampulla, percutaneous transhepatic external drainage is firstly recommended. The biliary stents are implanted after the biliary tract infection is improved.

#### 2.2.2. Pancreatic Duct Visualization

Compared with the percutaneous transhepatic route, endoscopic procedures in the biliary tract are more likely to cause acute pancreatitis and hyperamylaseemia [33, 34]. PTBS is more suitable than ERCP for patients with complex types of biliary obstruction [35]. However, acute pancreatitis after the percutaneous intervention is closely related to internal and external biliary drainage and stent placement. During the biliary intervention, a drainage catheter or stent across orifice of the pancreatic duct will affect the excretion of pancreatic juice. Meanwhile, the contrast agent, bile, duodenal juice, and pancreatic juice would flow back into the pancreatic duct, resulting in increased intraductal pressure. As a result, the retrograde bacterial infection would cause pancreatitis [36]. In addition, the clinicians' operational factors may also lead to acute pancreatitis during operation.

Pancreatic duct visualization during PTCD and PTBS is mainly due to cholangiography when the drainage catheter is at the distal end of the bile duct or is withdrawn through the papilla of Vater. Unlike the injection of contrast into the pancreatic duct during ERCP, this kind of visualization usually indicates pancreaticobiliary maljunction (PBM) or sphincter of Oddi dysfunction (SOD) in patients. They may increase the risk of postoperative pancreatitis. PBM is a congenital deformity in which the pancreatic and bile ducts unite anatomically outside the duodenal wall to form a long common channel [37]. It contributes to pancreatitis by causing a temporary or persistent obstruction of pancreatic juice or bile reflux into the pancreatic duct (biliopancreatic reflux) [38], followed by increased pressure in the pancreatic duct. A study found that PBM was one of the pathogenic factors of pancreatitis [39]. Kamisawa et al. believed that PBM caused biliopancreatic reflux, which increased the risk of pancreatitis and cholangiocarcinoma [40]. Thus, there are two main reasons for pancreatitis after pancreatic duct visualization during PTCD and PTBS. On the one hand, it may be related to the high pressure in the pancreatic duct caused by the injection of a contrast agent. However, more possibly, it is the result of the ampulla's abnormal anatomical structure and function. Therefore, when undertaking percutaneous intervention in patients with distal MBO, cholangiography with excessive pressure near the pancreatic orifice would be avoided. At the same time, it is also necessary to observe whether the ampulla's structure and function are abnormal after pancreatic duct visualization.

In terms of the endoscopic approach, pancreatic duct visualization is often a manifestation of excessive pressure caused by contrast injection into the pancreatic duct. It is also a relatively straightforward risk factor for PEP. Previous studies found that contrast injection into the pancreatic duct was an independent predictor of pancreatitis after endoscopic SEMS implantation [26, 41, 42]. The mechanism of this pancreatitis is that it increases the hydrostatic pressure in the pancreatic duct and leads to pancreatic duct edema and damage to the pancreatic duct wall and acinar epithelium [43]. This results in the high-pressure pancreatic juice flowing back into the parenchyma and finally activating pancreatin. Furthermore, pancreatic duct hypertension interferes with pancreatic juice secretion, and then the zymogen granules blend with lysosomes, which activate pancreatin in advance and ultimately lead to pancreatitis.

Previous studies suggested that indomethacin administered through the rectum before or after ERCP had an excellent preventive effect on PEP. It has been recommended by many guidelines, including the European Society of Gastrointestinal Endoscopy [44, 45]. In addition to indomethacin, pancreatic enzyme inhibitors can theoretically prevent postoperative pancreatitis because they can inhibit pancreatic exocrine function, and, thereby, reduce the level of self-digestion of the pancreas [46]. Similarly, pancreatic enzyme inhibitors used before PTCD and PTBS may also reduce postoperative blood amylase levels and the incidence of pancreatitis. For patients with PBM or SOD suggested by pancreatic duct visualization, the study of Yang et al. indicated that postoperative prophylactic application of somatostatin could restore partial pancreatic function and reduce significant complications [11].

#### 2.2.3. Stent and Drainage Catheter Implantation Position

PTCD includes simple external drainage, internal drainage, and internal–external drainage. It is generally believed that bile can flow into the duodenum during internal drainage, participate in food digestion, and complete the enterohepatic circulation of bilirubin, which is more consistent with physiological needs than simple external drainage [47]. Therefore, internal drainage is more clinically preferred for patients with distal biliary obstruction, where most lesions occur near the duodenal papilla. One end of the stent would be placed into the duodenum in these patients, ensuring better bile drainage after the transampullary papillary procedure [48, 49].

However, in previous studies, acute pancreatitis after biliary intervention occurred in patients who undertook biliary stents [50–54] or internal drainage catheters [55, 56] across the ampullary, which were not reported in patients who received external drainage alone. Recently, Sugawara et al. reported that biliary metal stent placement across Vater's papilla resulted in 24.2% of patients with acute pancreatitis requiring medical treatment [9]. In a retrospective study comparing the complications with SEMSs placed above or across the sphincter of Oddi in MBO, it was found that the incidence of acute pancreatitis in the suprapapillary stent group (4.1%) was lower than that in the transpapillary stent group (25.0%; *p* < 0.001) [57].

Acute pancreatitis caused by internal drainage catheters or biliary stents across the duodenal papilla may be related to the following factors: (1) clinician injures local pancreas during PTCD or PTBS produce; (2) biliary stents and internal drainage catheters compress or stimulate the duodenal papilla, which leads to increase in the pressure in the pancreatic duct and affects pancreatic juice discharge; (3) internal drainage catheters or stents obstruct the pancreatic duct orifice; (4) duodenal juice and bile flow back into the pancreatic duct through the side holes of internal drainage catheters or stent mesh; and (5) one end of the internal drainage catheter or stent in the descending segment of the duodenum will compress peripheral blood vessels and reduce blood supply to the pancreas [58]. Therefore, simple external drainage or suprapapillary stent implantation should be considered for patients with lesions greater than 2 cm from the Vater's ampulla [57].

## 3. Possible Risk Factors

### 3.1. Patient-Related Factors

Although, in previous studies, the influence of the patient's general condition was not adequately reported in acute pancreatitis after PTCD and PTBS, PEP-related studies showed that female, age (<40 years), and high body mass index (BMI) might be the reasons [16, 41, 42]. Female was a risk factor for PEP because SOD was more common in women [59]. Furthermore, SOD was considered a definite cause of acute pancreatitis [60]. SOD can obstruct the bile into the intestine and increase pressure in the bile duct, which can cause bile to flow back into the pancreatic duct and develop acute pancreatitis. Previous studies showed fat cell infiltration in pancreatic tissue after age 40 years, suggesting that the atrophy or disappearance of pancreatic tissue developed with age [61]. Sato et al. found that characteristic changes of pancreatic atrophy, lobulation, and steatosis were age-related in MRI [62]. Therefore, the differences in the incidence due to age may be because the pancreatic parenchyma is abundant and the pancreatic secretory function is more vigorous in younger patients [24]. In addition, obesity promotes inflammation and inhibits autophagy, which creates an environment that induces and contributes to pancreatitis progression [63]. This theory explains why increased BMI is a risk factor for PEP.

The most common pathogenic factors of common acute pancreatitis are gallstones and alcoholism. Others include hypercalcemia, hypertriglyceridemia, infection, genetics, and autoimmune diseases [15]. Metabolic causes, such as moderate hypertriglyceridemia (2–10 mmol/L or 177–886 mg/dL), may raise the risk of acute pancreatitis. High blood pressure, fatty liver, and diabetes may increase the severity of pancreatitis [64]. Whether these factors are also involved in acute pancreatitis after the biliary intervention is unknown.

For acute pancreatitis after an interventional operation, the inherent factors in patients may partly affect the occurrence of pancreatitis. Although they are not definite independent influence factors, further research must consider their impact when combined with other factors.

### 3.2. Stent Types

The types of biliary stents mainly include metal and plastic stents. The plastic stent is cheap and easy to operate and replace, but the diameter is small, and the long-term patency rate is poor. Generally, it needs to be replaced every three months. Metal stents include bare and covered stents, and SEMSs are mainly used in clinical practice. Compared with plastic stents, SEMS has better expandability and longer term patency [65–67]. However, many clinical studies on PEP showed that metal stents were more likely to cause acute pancreatitis than plastic stents. Several retrospective studies indicated patients with SEMS (6–17.6%) had a significantly higher incidence of pancreatitis than those with plastic stents (0–3.6%) [41, 68, 69] and an increased risk of postoperative pancreatitis (HR = 2.766; 95% CI, 1.348–5.677; *p* = 0.006) [42].

However, there were few reports that the type of stent influenced the incidence and severity of acute pancreatitis after PTBS. Previous studies compared bare metal stents with covered metal stents and the difference in stent diameters or lengths. The results showed no significant difference in the risk of acute pancreatitis between the type of stent [9, 11]. Isayama et al. found no significant difference in the incidence of acute pancreatitis complications after using bare metal and covered metal stents [70]. Two meta-analyses also reported that for patients with MBO, there was no difference in the incidence of acute postoperative pancreatitis between bare metal and covered metal stents (OR = 1.07; 95% CI, 0.44–2.59) [71] or SEMSs and plastic stents (OR = 0.93; 95% CI, 0.48–1.80) [72].

Some scholars believed that the biliary stent might compress the main pancreatic duct orifice or the common channel of the pancreaticobiliary duct, which resulted in different degrees of obstruction of the pancreatic duct [41]. This situation might be more serious with metal biliary stents than plastic stents because of the greater diameter and expansive radial force (RF) [42, 68, 69]. Itoi et al. proposed two main mechanisms for acute pancreatitis after SEMS implantation. One was the injection of a contrast agent into the main pancreatic duct during operation, and the other was that the compression of the papilla by the radial expansion force of SEMS affected the flow of pancreatic juice in the main pancreatic duct [73]. Martinez et al. believed that the main pancreatic duct dilation in patients with MBO might reduce the incidence of pancreatitis after stent implantation. The difference in the incidence of acute pancreatitis because of the stent type may be insignificant for these patients [74]. The relationship between the type of stent and acute pancreatitis after PTBS still needs further exploration and research.

### 3.3. Stent Mechanical Properties

The mechanical properties of SEMS mainly include axial force (AF) and AF is the restoring force to straighten the stent after bending, related to conformability in the bile duct. RF is the expansion force contributing to the luminal patency against the stricture [70, 75].

Some studies on PEP reported that high AF stents were closely related to an increased incidence of postoperative pancreatitis. It was speculated that the AF might compress the pancreatic duct orifice during the longitudinal deformation of the bile duct by the stent [14, 23]. Although SEMSs had a larger diameter and longer patency than plastic stents, it should be noted that the high AF SEMS was significantly associated with postoperative pancreatitis. A low AF SEMS better fitted the curved bile duct and did not interfere with stent patency [76, 77].

From the stent-related factors, the only difference between the percutaneous intervention and ERCP is the approach to stent implantation. Regardless of the stent type or mechanical properties after stent implantation, their effects on the occurrence of acute postoperative pancreatitis should be the same. However, this speculation needs to be confirmed in future clinical trials, and further research about the percutaneous intervention is still required.

## 4. Conclusions and Future Perspectives

Many studies on operation related complications have been conducted in recent years as more clinicians have chosen ERCP to treat distal MBO. Compared with the retrograde process in ERCP, the anterograde operation in the percutaneous interventions might reduce the damage because it conforms to the physiological flow of bile and the direction of bile duct movement. However, acute pancreatitis often occurs during this procedure. Current clinical studies showed that the probability of pancreatitis after PTCD and PTBS might be relatively low when pancreatic duct dilatation and pancreatic parenchyma atrophy were found in preoperative imaging. Visualizing the pancreatic duct, selecting internal drainage, and placing a biliary stent across the duodenal papilla might increase the incidence of pancreatitis to a certain extent. Besides the above factors, many uncertain and unknown factors might cause acute pancreatitis after the percutaneous intervention, such as certain factors leading to common acute pancreatitis and PEP. The reason may be a single factor or the superposition effect of multiple risk factors. Some risk factors may be because of short-term stimulation, and some may result from long-term accumulation. The occurrence and development of this acute pancreatitis may also involve a variety of pathogenic mechanisms. Without more high-level evidence-based medical evidence, the risk factors associated with common acute pancreatitis and PEP need to be noticed at this stage. In clinical practice, it is recommended to comprehensively estimate the risk of acute pancreatitis according to the patient's condition, primary disease, and obstruction site, and avoid adding multiple possible risk factors. For high-risk patients, simple percutaneous external drainage should be first considered to reduce local stimulation of the duodenal papilla, followed by PTBS may be safer.

In the future, more clinical studies will be conducted to provide high-level evidence to verify and objectively quantify these risk factors, facilitate the development of individualized treatment strategies, and decrease the incidence and mortality of acute pancreatitis after percutaneous interventional procedures.

## Data Availability

The data supporting this review article are from previously reported studies and datasets, which have been cited. The processed data are available from the corresponding author upon request.

## Abbreviations

PTCD: Percutaneous transhepatic cholangiodrainage
PTBS: Percutaneous transhepatic biliary stenting
MBO: Malignant biliary obstruction
ERCP: Endoscopic retrograde cholangiopancreatography
PEP: Post-endoscopic retrograde cholangiopancreatography pancreatitis
SEMS: Self-expandable metallic stent
PBM: Pancreaticobiliary maljunction
SOD: Sphincter of Oddi dysfunction.
